# Chronic subacute bowel obstruction caused by carcinoid tumour misdiagnosed as irritable bowel syndrome: a case report

**DOI:** 10.1186/1757-1626-2-78

**Published:** 2009-01-22

**Authors:** Henrietta M Wilson

**Affiliations:** 1 Department of Oncology, Royal Surrey County Hospital, Egerton Road, Guildford, Surrey, uk

## Abstract

**Background:**

Carcinoid tumours are well-differentiated neuroendocrine tumours with secretory properties. Although fairly rare, they are the most common malignancy seen to affect the distal small bowel. Presentation is often non-specific with symptoms mimicking those of irritable bowel syndrome. Given this, the condition is often diagnosed late following disease progression, by which time the prognosis is poor.

**Case presentation:**

A 74 year old Caucasian lady presented with a two week history of loose stools, nausea and one episode of vomiting. This sub-acute presentation occurred on a background of a four year history of intermittent abdominal pain and bloating, previously diagnosed as irritable bowel syndrome. CT scans identified dilated loops of small bowel proximal to a spiculated mass in the region of the terminal ileum. This ileal lesion was removed at laparotomy and identified as a carcinoid tumour.

**Conclusion:**

This case highlights the issue of misdiagnosis of intestinal malignancy as the benign condition of irritable bowel syndrome. There have been several other references to this happenstance in the literature, and the problem is reflected in the percentage of patients with widespread disease at the time of diagnosis. Prognosis in this condition can be dramatically improved with early diagnosis, and surgical management at this stage is often curative. For this reason it is imperative to keep this differential diagnosis in the back of one's mind when assessing patients presenting with symptoms of intermittent partial bowel obstruction. The clinical presentation of this tumour, along with investigation and management of these cases, is discussed here.

## Background

Midgut carcinoids are generally well-differentiated neuroendocrine tumours with secretory characteristics. An occurrence rate within the small intestine of 1 in 300 has been reported at autopsy, making it the most common distal small bowel malignancy [1]. In addition, when considering tumours of the jejunum and ileum, multicentricity occurs in 26–30% of cases [2]. These are also the most common tumours affecting the appendix accounting for 80% of appendiceal growths; however these are usually small, non-metastatic and cured by simple appendicectomy [3]. There is also a low incidence of metastasis from tumours within the small bowel; in spite of this, liver metastases are often present at the time of diagnosis as patients are identified late in the disease [4].

## Case presentation

A 74 year old lady presented to the emergency department following a fall. On further questioning she reported a two week history of loose stools, mild abdominal pain and one episode of vomiting. These symptoms occurred on a four year background of intermittent abdominal pain and bloating for which she had been referred to a surgical outpatient clinic three years previously. Following a normal gastroscopy and colonoscopy she was diagnosed with irritable bowel syndrome.

**Figure 1:**
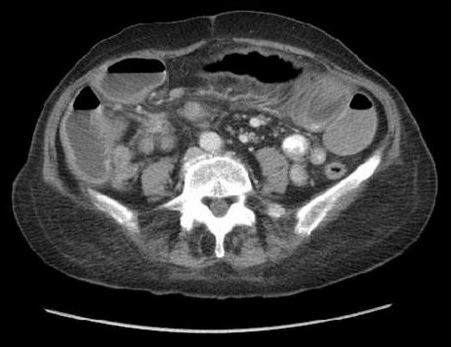
**Abdominal CT scan demonstrating dilated loops of bowel, a spiculated lesion in the terminal ileum and mesenteric lymph node enlargement**.

**Figure 2:**
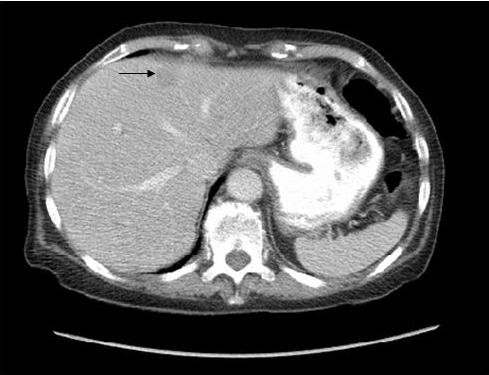
**Abdominal CT scan showing liver lesion**.

Physical examination showed abdominal distension with generalised tenderness and active bowel sounds. She was clinically dehydrated and blood tests revealed a low potassium of 2.9 and elevated urea of 12.9. The patient underwent an abdominal CT scan which identified dilated loops of small bowel proximal to a spiculated mass in the region of the terminal ileum with mesenteric lymph nodes. Multiple small lesions within the liver were also noted. [Figure 1: CT showing dilated loops of bowel, spiculated lesion and mesenteric lymph node enlargement. Figure 2: CT showing liver lesion (arrow)]

At laparotomy the surgical team located the lesion within an ischaemic terminal ileum. They also found significant mesenteric lymphadenopathy. The team carried out a small bowel resection with ileo-caecal anastomosis. At histology the lesion within the terminal ileum was identified as a carcinoid tumour measuring 2 cm, with mesenteric nodal metastasis in the proximal ileum. The patient made a good post-operative recovery and went on for further assessment to identify metastatic disease.

## Discussion

The term carcinoid was first used to describe 'hormonally active' tumours in 1907 by Oberndorfer [5]. They are derived from stem cells in the gut wall and have been classified depending on the location within the primitive gut from which they arise [6]. Tumours of the bronchus, stomach, proximal duodenum and pancreas are derived from the foregut; the midgut gives rise to tumours found in the second portion of the duodenum, the jejunum, the ileum and the right colon, while those of the hindgut include the transverse colon, descending colon, and rectum. Given the tumour site in this case, further discussion will refer to midgut tumours only.

Clinical presentation can be with non-hormonal symptoms secondary to tumour bulk and local reaction, or with symptoms of a functioning tumour, described as 'carcinoid syndrome'. Tumours arising in the small intestine are often silent until late in the disease and are thus locally advanced at presentation [7]. Non-hormonal symptoms are most commonly secondary to partial mechanical obstruction of the small bowel with patients complaining of intermittent vague abdominal symptoms such as pain and distention. It is for this reason that patients with carcinoids are frequently misdiagnosed with irritable bowel syndrome [1]. These obstructive features may be due to peritumoral fibrosis or invasion, causing direct luminal strictures, or secondary to desmoplastic reaction leading to ischaemic changes [8]. Other non-hormonal symptoms include anorexia, weight loss, fatigue and occasionally a palpable abdominal mass is noted.

**Figure 3:**
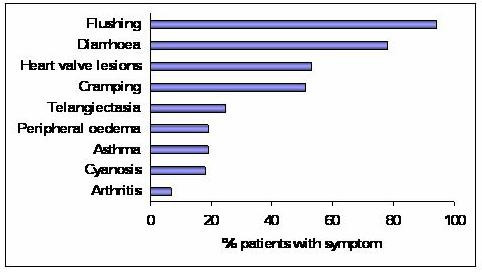
**Bar chart plotting the prevalence of symptoms and signs occurring in carcinoid syndrome**. Data taken from http://www.carcinoid.com

The term 'carcinoid syndrome' is used to describe the hormonal manifestations of carcinoid tumours and occurs secondary to the secretion of serotonin, tachykinins, bradykinins and prostaglandins. Midgut carcinoid tumours are typically seen to secrete serotonin [9]. Recent consensus is that functionally active tumours account for around 20–30% in this group, higher than previously thought [4]. The syndrome occurs when vasoactive substances from tumour secretion enter the systemic circulation. In the case of small bowel tumours this can only occur in the presence of multiple hepatic metastases, as substances such as serotonin usually undergo hepatic degradation to non-active substrates. The symptoms of carcinoid syndrome and their prevalence are outlined in figure 3: Prevalence of symptoms occurring in carcinoid syndrome.

Investigation of these symptoms is best done with a multimodality approach including biochemical investigation, radiological and nuclear imaging, and finally histological confirmation where possible. In patients presenting with symptoms of carcinoid syndrome urinalysis can be carried out for elevated 5-HIAA levels. Chromogranin A can also be used as a sensitive marker to identify midgut carcinoid tumours, however, specificity may be low [10]. The most sensitive imaging techniques depend on the site of the tumour but may include CT, MRI and ultrasound scanning in combination with endoscopy and small bowel studies. Where a gastrointestinal neuroendocrine tumour is suspected or confirmed, somatostatin receptor scintigraphy is routinely used to locate both primary and secondary deposits for staging of the disease. Somatostatin receptor type 2 is expressed by 80% of carcinoid tumours allowing this mode of imaging a sensitivity of up to 90% for midgut tumours [11].

Given that the majority of these tumours are well-differentiated, following an indolent course, prognosis is surprisingly unfavorable. This can mainly be attributed to the fact that the majority of cases present so late by which time spread has invariably occurred. Survival correlates closely to stage at presentation with a 5-year survival of 65% reported for localized/regional disease and 36% if distant metastases are present [4].

The principal management approach in these cases is surgical resection of the primary lesion and is the only curative option. With smaller lesions (< 1 cm) local resection is usually adequate. However, with lesions over 1.5 cm there is a high risk of recurrence and thus segmental resection is required with extensive clearance of the associated mesenteric lymph nodes [1]. Surgery has been shown to be of benefit even in patients with metastatic disease, both to gain symptomatic relief and improve survival [4].

Further management of patients with liver metasteses is still controversial. Treatment options include resection of the hepatic tumours, or hepatic artery embolisation (HAE) as an alternative to surgery. There are a number of studies in the literature looking into the benefits of hepatic resection with regards to both symptomatic relief and prolonged survival rates. Que et al. report a 90% symptomatic response with liver resection and state that debulking extends survival; however recurrence is expected [12]. Other authors have also reported the possibility of a prolonged survival time, but it should be pointed out that in this study the comparison group were those patients with inoperable disease who may have therefore had more advanced underlying disease [13]. Similar benefits have been demonstrated using HAE with palliation of both hormonal and pain symptoms [14]. Although advantages have been seen with these approaches it should be noted that, given the rare nature of this condition, population groups within the research have been small and studies have generally been retrospective.

Other possibilities for symptomatic management of patients with hormonal symptoms include biotherapy using drugs such as somatostatin analogs. These have been shown not only to provide symptomatic improvement in 70–80% but also to stabilize tumour growth [4]. This therapy is therefore used as first-line treatment in inoperable functioning tumours and to avoid a carcinoid crisis in those undergoing surgery.

## Conclusion

Despite the rarity of this condition, carcinoid tumours are the most common lesion seen to affect the distal small intestine. Unfortunately, in the majority of cases presentation is often late in the disease and metastases are therefore regularly seen at the time of diagnosis. Analysis of five-year survival rates has identified several factors associated with a poor prognosis including multiple liver metasteses, the presence of carcinoid syndrome and high levels of hormonal markers [15]. Given that the clinical presentation is often with non-specific abdominal symptoms, diagnosis can be difficult and patients are frequently misdiagnosed with irritable bowel syndrome [1]. Surgical resection is the only curative management option and there is evidence to support a better prognosis when lesions are small. Consequently it is important to keep this differential diagnosis in the back of one's mind when assessing patients presenting with symptoms of intermittent partial bowel obstruction to improve early diagnosis and outcomes in this patient group.

## Consent

Written informed consent was obtained from the patient for publication of this case report and accompanying images. A copy of the written consent is available for review by the Editor-in-Chief of this journal.

## Competing interests

The author declares that they have no competing interests.
